# Head to head comparison of Prasugrel versus Ticagrelor in patients with acute coronary syndrome: a systematic review and meta-analysis of randomized trials

**DOI:** 10.1186/s40360-017-0189-7

**Published:** 2017-12-12

**Authors:** Pravesh Kumar Bundhun, Jia-Xin Shi, Feng Huang

**Affiliations:** 1 Institute of Cardiovascular Diseases, the First Affiliated Hospital of Guangxi Medical University, Nanning, Guangxi 530027 People’s Republic of China; 2 Institute of Cardiovascular Diseases and Guangxi Key Laboratory Base of Precision Medicine in Cardio-cerebrovascular Diseases Control and Prevention, the First Affiliated Hospital of Guangxi Medical University, Nanning, Guangxi 530021 People’s Republic of China

**Keywords:** Prasugrel, Ticagrelor, Acute coronary syndrome, Anti-platelets, Clinical outcomes, Meta-analysis

## Abstract

**Background:**

Prasugrel and Ticagrelor are emerging antiplatelet drugs that might have the potential to replace currently used antiplatelet agents. Previous analyses comparing prasugrel with ticagrelor mainly focused on an indirect comparison whereas direct comparison was reported only in a few recently published trials. We aimed to systematically carry out a head to head comparison of the adverse clinical outcomes which were associated with prasugrel versus ticagrelor in patients with acute coronary syndrome (ACS).

**Methods:**

Studies comparing prasugrel with ticagrelor (head to head comparison) were searched from online databases. Adverse cardiovascular outcomes were considered as the primary endpoints whereas bleeding outcomes were considered as the secondary endpoints in this analysis. The latest version of the RevMan software was used to carry out subgroup analyses whereby odds ratios (OR) with 95% confidence intervals (CI) and the calculated probability (P) were generated.

**Results:**

Four studies with a total number of 563 patients (2012 – 2016) were included (282 patients were treated with prasugrel and 281 patients were treated with ticagrelor). Results of this analysis did not show any significant difference in mortality between prasugrel and ticagrelor with OR: 1.52, 95% CI: 0.42 – 5.45; *P* = 0.52. In addition, myocardial infarction, major adverse cardiac events, stroke and stent thrombosis were also not significantly different with OR: 0.59, 95% CI: 0.08 – 4.58; *P* = 0.62, OR: 0.91, 95% CI: 0.37 – 2.21; *P* = 0.83, OR: 0.60, 95% CI: 0.08 – 4.58; P = 0.62 and OR: 0.59, 95% CI: 0.08 – 4.58; P = 0.62 respectively.

Thrombolysis in myocardial infarction (TIMI) defined minor bleeding, and minimal bleeding were also not significantly different between these two newer antiplatelet agents with OR: 3.11, 95% CI: 0.48 – 19.94; *P* = 0.23, and OR: 2.39, 95% CI: 0.35 – 16.42; *P* = 0.38 respectively. Moreover, bleeding defined by the academic research consortium was also similarly manifested with OR: 0.92, 95% CI: 0.39 – 2.13; *P* = 0.84.

**Conclusion:**

In patients with ACS, both prasugrel and ticagrelor showed similar adverse cardiovascular outcomes and bleeding events. No significant difference was observed between these two newer antiplatelet agents during this head to head comparison. However, upcoming trials with long term follow up periods might be expected to completely solve this important clinical issue.

## Background

Prasugrel and Ticagrelor are newer emerging antiplatelet drugs which might have the potential to replace currently used antiplatelet agents [1, 2]. However, even if comparison of these two drugs is very important clinically, this has often been a controversial issue. The only network meta-analysis that has been published until date suggested that both prasugrel and ticagrelor were clinically effective when they were compared to clopidogrel, and when they were indirectly compared to each other, the former showed to be more effective in preventing recurrent ischemic episodes and stent thrombosis [3]. However, we believe that this indirect comparison might absolutely not generate standard results as compared to a direct head to head comparison. To support this point, Morici et al. pointed out that any indirect comparison between prasugrel and ticagrelor would be impossible based on the different patients’ populations and unequal clinical settings rendering the attempt to find specific usage of these two antiplatelet drugs in different clinical settings to appear questionable [4].

Therefore, the focus is now on the upcoming Intracoronary Stenting and Antithrombotic Regimen: Rapidly Early Action for Coronary Treatment (ISAR-REACT) 5 Trial which is believed to effectively compare these two newer antiplatelet drugs clinically [5].

However, in order to shorten the gap, we aimed to systematically carry out a head to head comparison of the adverse clinical outcomes which were associated with prasugrel versus ticagrelor in patients with acute coronary syndrome (ACS), through a meta-analysis of recently published randomized cohorts.

## Methods

### Data sources, searched terms and strategies

Online data sources: MEDLINE/PubMed, EMBASE and the Cochrane databases. Publications comparing prasugrel with ticagrelor (head to head comparison) were searched using the terms ‘prasugrel and ticagrelor’. In addition to the previously mentioned terms, other words or abbreviations such as: coronary interventions, percutaneous coronary intervention, coronary angioplasty, acute coronary syndrome, PCI, ACS, newer oral antiplatelet agent’ were also used in the searched strategy. This search was restricted to articles which were published in English language.

### Inclusion criteria

Studies were included if:

- They were randomized trials comparing (head to head) prasugrel with ticagrelor.
- They reported at least one adverse clinical outcome as their endpoint.
- They consisted of patients with ACS or patients who underwent percutaneous coronary intervention (PCI).

### Exclusion criteria

Studies were excluded if:

- They were meta-analyses, case studies or randomized trial/observational studies indirectly comparing prasugrel with ticagrelor (not head to head comparison).
- They only reported platelet activities without reporting any adverse clinical endpoint.
- They were duplicates or they involved the same trial.

### Endpoints assessed

The primary endpoints were:

- Major adverse cardiac events (MACEs) consisting of death, myocardial infarction (MI) and stroke or any of the below mentioned outcomes;
- Mortality (cardiac and non-cardiac death);
- Cardiac death;
- Stent thrombosis (ST);
- Stroke;
- MI.

The secondary endpoints were:

- Any bleeding (major or minor or minimal);
- Thrombolysis in myocardial infarction (TIMI) defined minor bleeding [6];
- TIMI defined major bleeding;
- TIMI defined minimal bleeding;
- Bleeding defined by academic research consortium (BARC) [7].

Table 1 summarized these outcomes with their corresponding follow up periods.

**Table 1:** Outcomes which were reported

Studies	Reported outcomes	Follow up periods
Bonello [10]	Cardiovascular death, stroke, MACEs, BARC bleeding	30 days
Laine [11]	Death, MACEs, bleeding	In hospital
Motovska [12]	MACEs, cardiovascular death, all-cause death, MI, stroke, ST, TIMI major, minor and minimal bleeding, BARC bleeding	7 days, 30 days
Parodi [13]	Death, MI, ST, TIMI major, minor, minimal bleeding, stroke, MACEs	In hospital

*Abbreviations*: *MI* myocardial infarction, *MACEs* major adverse cardiac events, *ST* stent thrombosis, *TIMI* thrombolysis in myocardial infarction, *BARC* bleeding defined by the academic research consortium

### Data extraction, quality assessment and review

Two authors (PKB and JXS) independently reviewed the final publications which were selected for this analysis. Their titles and abstracts were carefully checked to ensure that they were completely relevant (head to head comparison only) and to be sure that they reported the correct endpoints which were later extracted and tabulated. Information and data involving the total number of participants who were treated by prasugrel and ticagrelor respectively, the period of patients’ enrollment (in years), the follow up periods (in hospital, number of days or months), the baseline characteristics (age, gender, co-morbidities), and the number of events which were reported in each group were carefully extracted independently by these same authors and cross-checked later to make sure that no wrong data or typing errors were introduced. Any disagreement, if present, was discussed with another author (FH) and a final decision was made. The PRISMA guideline was followed [8]. Bias risk was also assessed (Cochrane Collaboration) [9] and an average rating (low, moderate or high-risk bias) was allotted to all the trials.

### Statistical analysis

The latest version of the RevMan software (version 5.3) was used during the subgroup analysis and the odds ratios (OR) with 95% confidence intervals (CI) as well as the calculated probability (*P* value) were generated. Heterogeneity [10] which was an integral part of the analysis, was assessed by two simple statistical methods: the (Q-statistic test) and the (I^2^ test) with reference to the following rules: (a) if the *P* value was less or equal to 0.05, the result was considered statistically significant and if the P value was more than 0.05, the result was considered insignificant, and (b) an increasing I^2^ would denote an increasing heterogeneity, therefore the lower the I^2^ value, the less heterogeneous would be the result. Moreover, a fixed (I^2^ < 50%) or a random (I^2^ > 50%) effects model was used based on the corresponding I^2^ value which was obtained.

To ensure that the results were not influenced by one particular trial, sensitivity analysis was also carried out. Each study was excluded one by one, and then a new analysis was carried out and compared with the results of the main analysis.

Moreover, publication bias which was expected in this analysis, was visually estimated by assessing the funnel plots which were obtained from the Revman software.

### Ethics

Ethical or board review approval was not necessary for this type of analysis.

## Results

### Study selection

A total number of one hundred and twenty-two (122) publications were obtained from electronic databases. After carefully assessing the titles and abstracts, ninety-seven (97) publications were eliminated since they were not completely relevant to this current research. Twenty-five (25) full text articles were assessed for eligibility. Further full text articles were eliminated since:

- One (1) article was a network meta-analysis;
- Three (3) articles were case studies;
- Six (6) studies only assessed platelet reactivity without reporting any clinical outcome;
- Eleven (11) studies were duplicates or involved the same trial.

**Fig. 1:**
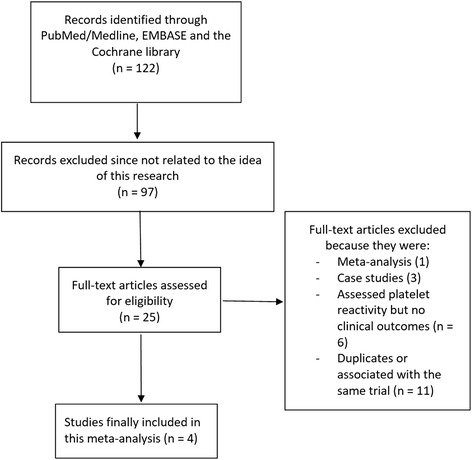
Flow diagram representing the study selection

Finally, only four (4) studies [11–14] were selected for this analysis. This study selection process has been represented in Fig. 1.

### General features of the studies which were included

Table 2 summarized the general features of the studies which were included in this analysis. All the studies were randomized trials with a total number of 563 patients (282 patients were treated with prasugrel and 281 patients were treated with ticagrelor). Patients’ enrollment period ranged from 2012 to 2016.

**Table 2:** General features of the trials

Studies	No of patients treated with prasugrel (*n*)	No of patients treated with ticagrelor (*n*)	Total no of patients (*n*)	Year of patients’ enrollment (years)	Type of study
Bonello	107	106	213	2014	RCT
Laine	50	50	100	2012 - 2013	RCT
Motovska	100	100	200	2013 - 2016	RCT
Parodi	25	25	50	–	RCT
Total no of patients (*n*)	282	281	563		

*Abbreviations*: *RCT* randomized controlled trials

### Baseline features of the studies which were included

Table 3 summarized the baseline features of the patients in both groups. A mean age ranging between 60 and 67 years was reported among the patients. Most of the participants were male patients with several co-morbidities such as hypertension, dyslipidemia and diabetes mellitus. With the exception of studies Motovska et al. and Laine et al., which included almost a similar number of patients with diabetes mellitus and who were treated by prasugrel or ticagrelor, the other two studies included more patients with diabetes mellitus in the prasugrel group. In other words, prasugrel was mainly reserved for patients with diabetes mellitus, whereas at baseline, all the other co-morbidities were almost equally distributed between the two groups (no significant difference).

**Table 3:** Baseline features of the participants

Studies	Age (years)	Males (%)	HT (%)	Ds (%)	Cs (%)	DM (%)
	P/T	P/T	P/T	P/T	P/T	P/T
Bonello	60.0/61.5	79.8/69.8	57.9/52.8	45.3/53.3	36.8/48.0	41.1/29.2
Laine	62.8/64.8	86.0/66.0	70.0/80.0	62.0/56.0	28.0/28.0	100/100
Motovska	61.8/61.8	77.1/73.7	51.4/51.2	33.4/35.4	64.0/65.8	20.0/20.8
Parodi	67.0/67.0	80.0/76.0	60.0/72.0	20.0/40.0	36.0/36.0	24.0/12.0

*Abbreviations*: *P* prasugrel, *T* ticagrelor, *HT* hypertension, *Ds* dyslipidemia, *Cs* current smoking, *DM* diabetes mellitus

### Primary outcomes

**Fig. 2:**
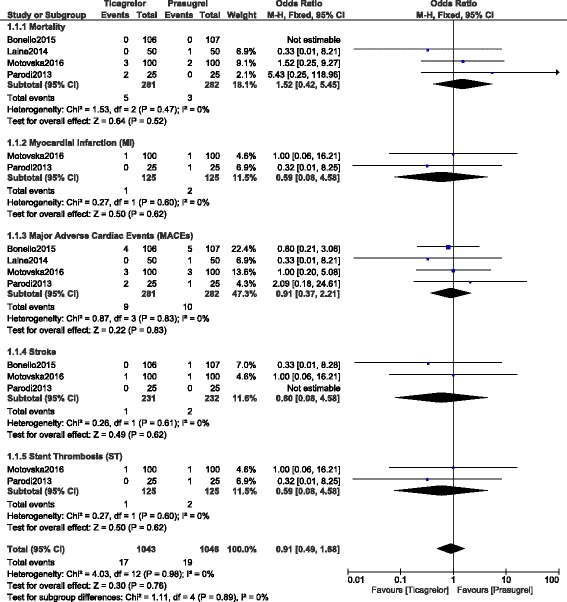
Primary outcomes which were observed between prasugrel and ticagrelor

Results of this analysis (Table 4) showed that mortality was not significantly different between prasugrel and ticagrelor with OR: 1.52, 95% CI: 0.42 – 5.45; *P* = 0.52, I^2^ = 0%. The current results also showed that MI, MACEs, stroke and ST were also not significantly different between prasugrel and ticagrelor with OR: 0.59, 95% CI: 0.08 – 4.58; *P* = 0.62, OR: 0.91, 95% CI: 0.37 – 2.21; *P* = 0.83, OR: 0.60, 95% CI: 0.08 – 4.58; P = 0.62 and OR: 0.59, 95% CI: 0.08 – 4.58; P = 0.62 respectively. Results involving the primary outcomes have been illustrated in Fig. 2.

**Table 4:** Results of this analysis

Outcomes analyzed	No of studies involved	OR with 95% CI	P value	I^2^ (%)
Mortality	4	1.52 [0.42 – 5.45]	0.52	0
MI	2	0.59 [0.08 – 4.58]	0.62	0
MACEs	4	0.91 [0.37 – 2.21]	0.83	0
Stroke	3	0.60 [0.08 – 4.58]	0.62	0
ST	2	0.59 [0.08 – 4.58]	0.62	0
Any bleeding	4	1.30 [0.64 – 2.64]	0.47	37
TIMI minor bleeding	2	3.11 [0.48 – 19.94]	0.23	1
TIMI minimal bleeding	2	2.39 [0.35 – 16.42]	0.38	0
BARC defined bleeding	2	0.92 [0.39 – 2.13]	0.84	0

*Abbreviations*: *MI* myocardial infarction, *MACEs* major adverse cardiac events, *ST* stent thrombosis, *TIMI* thrombolysis in myocardial infarction, *BARC* bleeding defined by the academic research consortium, *OR* odds ratios, *CI* confidence intervals

### Secondary outcomes

**Fig. 3:**
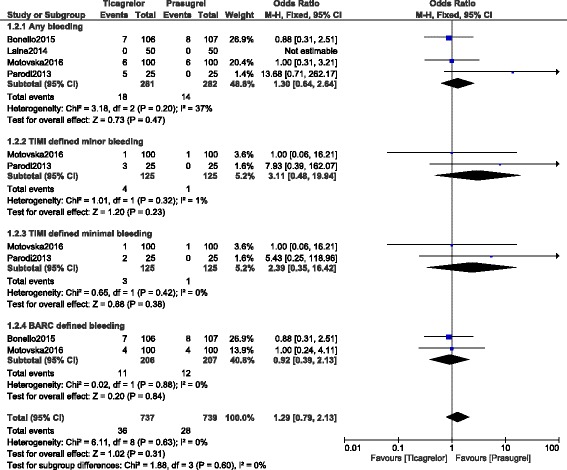
Secondary outcomes which were observed between prasugrel and ticagrelor

When the bleeding outcomes were analyzed, ‘any bleeding’ which consisted of a combination of major, minor and minimal bleeding was not significantly different between prasugrel and ticagrelor with OR: 1.16, 95% CI: 0.76 – 1.76; *P* = 0.50, I^2^ = 35%. TIMI defined minor bleeding, and minimal bleeding were also not significantly different with OR: 3.11, 95% CI: 0.48 – 19.94; *P* = 0.23, and OR: 2.39, 95% CI: 0.35 – 16.42; *P* = 0.38 respectively. In addition, BARC defined bleeding was also similarly manifested with OR: 0.92, 95% CI: 0.39 – 2.13; *P* = 0.84, I^2^ = 0%. Results involving the secondary outcomes have been represented in Fig. 3.

### Sensitivity analysis

When study Bonello et al. was excluded, no significant difference was observed in mortality, MACEs (OR: 0.95, 95% CI: 0.49 – 1.82; *P* = 0.87) or the other outcomes. When study Laine et al. was excluded and a new analysis was carried out, a similar result was obtained for mortality and MACEs with OR: 1.36, 95% CI: 0.68 – 2.73; *P* = 0.39 and OR: 0.96, 95% CI: 0.52 – 1.74; *P* = 0.88 respectively and when study Motovska et al. was excluded and a new analysis was carried out, no significant difference was observed among the endpoints with mortality and MACEs reporting OR: 1.52, 95% CI: 0.25 – 9.25; *P* = 0.65 and OR: 0.87, 95% CI: 0.30 – 2.53; *P* = 0.79 respectively. Excluding study Parodi et al. also did not affect/influence the main results of this current analysis whether for the primary or the secondary outcomes. In brief, based on this sensitivity analysis, consistent results were continually obtained.

### Publication bias

**Fig. 4:**
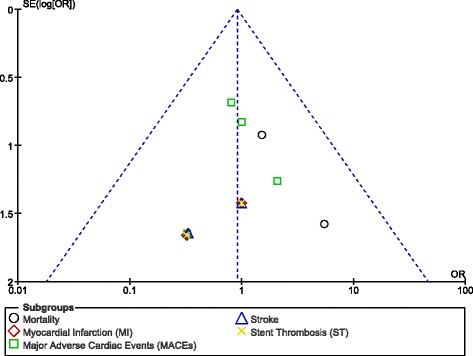
Funnel plot showing publication bias (A)

**Fig. 5:**
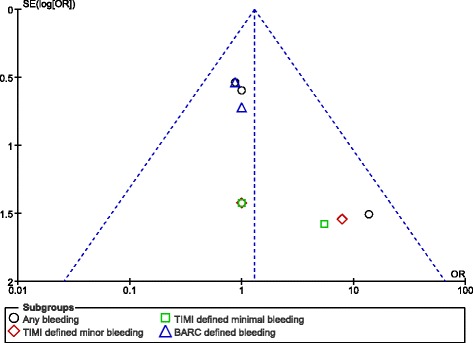
Funnel plot showing publication bias (B)

After visually assessing the funnel plots which were obtained, there was very little evidence of publication bias which was observed among the trials that assessed all the primary and secondary clinical endpoints in this analysis. These funnel plots have been illustrated in Figs. 4 and 5.

## Discussion

Clopidogrel is still considered as one of the most appropriate anti-platelet agent which is used along with aspirin post PCI. However, prasugrel and ticagrelor are newer emerging antiplatelet drugs which might be more potent in comparison to clopidogrel. Even if the mechanism of action is almost similar, prasugrel is associated with a more rapid and consistent inhibition of platelet in comparison to clopidogrel [15]. Recent studies have shown the effect of clopidogrel to have been reduced with proton pump inhibitor [16]. However, no such effect has been observed with prasugrel. Ticagrelor is also fast-acting and consistent in action when compared to clopidogrel, and it is associated with a rapid platelet function recovery [15].

Considering the fact that little research has been done to address such an important issue which is new in clinical medicine as well, and considering its controversial aspect, we aimed to compare (head to head comparison) prasugrel versus ticagrelor in patients with ACS.

Results of this analysis showed similar adverse cardiovascular (primary) and bleeding (secondary) outcomes observed between these two newer potential antiplatelet agents. No significant statistical difference was observed in any of the subgroup of outcomes which were analyzed.

Are the mechanisms of action of prasugrel and ticagrelor also similar? This might be a question which should be debated following the current results which were obtained. First of all, prasugrel, which is similar to clopidogrel in action, and which is a pro drug requiring metabolic activation, works by directly and irreversibly blocking the ADP-binding site of P2Y12 receptor leading to a permanent inhibition [17]. In contrast, ticagrelor, which does not require metabolic activation, reversibly prevents binding of ADP to P2Y12 receptors [18]. This is how these two anti-platelets work.

Similar to the results of this current analysis, the PRAGUE 18 study which consisted of 1230 patients also showed no superiority of one antiplatelet to the other (referring to prasugrel and ticagrelor only) in these patients with ACS [13]. However, the authors concluded that even if the major outcomes were similar between prasugrel and ticagrelor, broad CIs were observed around the estimates, which would require to be confirmed in larger trials. Moreover, the Rapid Activity of Platelet Inhibitor Drugs (RAPID) Primary PCI Study also showed non-inferiority of prasugrel with ticagrelor further supporting these current results [14]. A recent network meta-analysis comparing cangrelor, clopidogrel, ticagrelor and prasugrel post-intervention showed both ticagrelor and prasugrel to be comparable in terms of clinical outcomes [19].

Furthermore, a nationwide long-term registry analysis from the year 2009 to 2014 which included 32, 830 patients in total, showed an increasing use of prasugrel and ticagrelor. These anti-platelets were associated with significantly lower re-infarction and mortality as compared to clopidogrel. However, clopidogrel was preferred in older patients with an increased number of co-morbidities [20].

Nevertheless, the network meta-analysis which was published by Chatterjee et al. in which, they indirectly compared prasugrel with ticagrelor showed prasugrel to be surprisingly more effective than ticagrelor in the prevention of ST and recurrent ischemic events [3]. Their results which showed ticagrelor to be inferior to prasugrel, or prasugrel to be superior to ticagrelor, might have been due to the fact that an indirect comparison was made (no head to head comparison), with less patients.

In this analysis, a general population of patients with ACS were analyzed. The current results showed both prasugrel and ticagrelor to be associated with similar clinical outcomes post intervention. However, in an analysis of a particular subgroup of patients with diabetes mellitus [21], ticagrelor showed to have reduced the occurrence of several adverse clinical outcomes as well as platelet reactivity without increasing the risk of any bleeding event. However, the authors concluded that cautions need to be taken with the use of ticagrelor in clinical practice. Also, other studies showed dyspnea and contrast-induced nephropathy to be higher with ticagrelor compared to prasugrel [13].

In contrast to this current analysis which dealt with the adverse clinical outcomes, other studies have compared platelet activities which were observed between prasugrel and ticagrelor. The study published by Perl et al. in 2015 showed that treatment with ticagrelor resulted in greater platelet inhibition when compared to prasugrel in patients with ACS [22]. Another pharmacodynamic study also showed prasugrel to have produced significantly lower platelet inhibition compared to ticagrelor [23]. However, it was a crossover study whereby both treatments were used alternatively by the same patients.

The main focus is now on the ISAR REACT 5 trial which is a multicenter, randomized, open-label phase IV trial that has been set up to assess/confirm the superiority/ inferiority or similar benefit of ticagrelor with prasugrel in patients with ACS (STEMI, NSTEMI, unstable angina) with planned invasive strategy [5]. Primary endpoints in this trial will be analyzed according to intention-to-treat principle, blinded strategies will be followed. Endpoints which will be assessed will be similar to the endpoints assessed in this current analysis, but with a longer follow up period. Clinical follow up will be scheduled at 30 days, 6 and 12 months and a longer follow up period of 3 years as from randomization for mortality. This trial, as well as other future upcoming trials might be expected to completely solve this issue.

### Novelty

This analysis is new in different ways:

- This idea is very new in clinical medicine.
- This analysis addresses an important issue concerning the treatment options in patients with ACS.
- This is the first meta-analysis with a head to head comparison between prasugrel and ticagrelor.
- A very low level of heterogeneity which was observed among all the subgroups analyzed might also be a new feature of this analysis.

### Limitations

Limitations were as follows:

- Even if a large number of patients were included compared to previously published studies, the total number of patients was not high enough to reach a strong conclusion.
- Because of the fact that all the studies which were included in this analysis did not report similar endpoints, many subgroups only involved two studies for comparison of the outcomes.
- This analysis had a shorter follow up period. Long term follow up period would have strengthened this manuscript.
- Inclusion of different types of patients, for example, a larger number of patients with diabetes mellitus which were treated with prasugrel might show an unequal distribution when compared to patients treated with ticagrelor.
- Moreover, in order for the results of this analysis not to be influenced by the results of study Motovska et al. which consisted of the majority of patients compared to the other studies which were included in this analysis, the total number of patients representing study Motovska et al. was reduced to 200 patients.

## Conclusions

In patients with ACS, both prasugrel and ticagrelor showed similar adverse cardiovascular outcomes and bleeding events. No significant difference was observed between these two newer antiplatelet agents during this head to head comparison. However, upcoming trials with long term follow up periods might be expected to completely solve this important clinical issue.

## Abbreviations

ACS: acute coronary syndrome
MACEs: major adverse cardiac events, ST = stent thrombosis
PCI: percutaneous coronary intervention
